# Investigation on Using SBS and Active Carbon Filler to Reduce the VOC Emission from Bituminous Materials

**DOI:** 10.3390/ma7096130

**Published:** 2014-08-26

**Authors:** Peiqiang Cui, Shaopeng Wu, Fuzhou Li, Yue Xiao, Honghua Zhang

**Affiliations:** State Key Laboratory of Silicate Materials for Architectures, Wuhan University of Technology, Wuhan 430070, China; E-Mails: cuipeiqiang@whut.edu.cn (P.C.); wusp@whut.edu.cn (S.W.); xiaoy@whut.edu.cn (Y.X.); huahongzhang_88@whut.edu.cn (H.Z.)

**Keywords:** volatile organic compounds (VOC), bituminous materials, ultraviolet-visible spectroscopy test, thermogravimetric analysis-mass spectrometry

## Abstract

Bituminous materials are playing a vital role in pavement design and the roofing industry because of outstanding properties. Unfortunately, bituminous materials will release volatile organic compounds (VOC), making them non-environmentally friendly. Therefore, technologies that can be used to decrease the VOC emission are urgently required. In this research, the VOC emission and material behaviors were analyzed and compared to investigate the possibility of adding styrene butadiene styrene (SBS) and active carbon filler into bituminous materials to develop environmentally-friendly materials. Thermal gravimetric analysis-mass spectrometry (TG-MS) and ultraviolet-visible spectroscopy testing (UV-Vis) were employed to characterize the VOC emission process. Temperature sweep testing and frequency sweep testing were conducted to evaluate the rheological properties of bituminous materials. Research results indicated that the combined introduction of 4 wt% styrene butadiene styrene (SBS) and 4 wt% active carbon filler cannot only significantly lower the VOC emission speed and amount, but also improve the deformation resistance behavior at a higher temperature. SBS and active carbon filler can be used to reduce the VOC emission form bituminous materials.

## 1. Introduction

During the construction process and service period, bituminous materials will release volatile organic compounds (VOC), which are harmful for the environment and contractors [1]. Such VOC emission would cause environmental pollutions and toxicity to humans and aquatic organisms [2,3]. An example cross-sectional study conducted by Gamble [4] and Karakaya [5] proved that workers exposed weekly to bitumen fume had more symptoms, such as eye irritation and abnormal fatigue.

VOC emission is not only harmful to pavement workers and the environment, but it also degrades the material behavior of bituminous materials. Therefore, innovative technologies of detection and analysis technology for VOC and the development of VOC inhibitors to reduce the emission speed and amount are becoming more and more important to achieve green asphalt pavement. Nowadays, a number of construction guidelines [6,7] are requiring bituminous materials with a lower VOC content. This is a common requirement, especially in regards to construction for environmentally-sensitive areas (e.g., living area, schools and wetlands). Gasthauer [8,9] developed a process to identify the VOC from bituminous materials with gas chromatography coupled with mass spectrometry. It was reported that the VOC emission depended on various parameters, such as the asphalt temperature, the asphalt oxidation, the humidity of air, and so on.

Styrene butadiene styrene (SBS) has very good compatibility with bitumen and was added into bituminous materials to improve the material behaviors for pavement construction and roofing purposes [10,11]. The SBS molecules dissolved in bituminous materials will swell and absorb the soft components of bitumen. This swell and absorption process not only makes it an excellent property modifier [12], but also it has a potential function to make it difficult for the smaller molecules inside of the bituminous materials to escape from the surface of the bitumen. This means that SBS might be used as an inhibitor to prevent the release of VOC. Activated carbon has extremely high surface energy and has a porous structure that offers a large specific surface. It is commonly used for removing organic constituents and residual disinfectants in water supplies. It is a favored water treatment technique because of its multifunctional nature. Research has also proven that active carbon filler can be added into bituminous materials to improve the material behaviors [13,14]. Therefore, active carbon filler also might have a strong capacity of adsorption to achieve the purpose of preventing VOC from releasing.

The aim of this work was to understand the possibility of using SBS and active carbon filler to reduce the VOC emission from bituminous materials, as well as the material behaviors of such VOC-inhibited bituminous materials. The VOC emission speed and amount from bituminous materials were studied by means of thermal gravimetric analysis-mass spectrometer testing and ultraviolet-visible spectroscopy testing, respectively. The rheological properties of bituminous materials modified by VOC-inhibitors, such as SBS and active carbon filler, were characterized with temperature sweep and frequency sweep analyses.

## 2. Materials and Test Methods

### 2.1. Raw Materials

Two kinds of bituminous materials, PJ-90 bituminous materials and modified bituminous materials, were used. A combination of 4 wt% of SBS and 4 wt% of active carbon filler were added into the bituminous materials to investigate the influence on the materials’ properties and VOC emission characteristics by adding such inhibitors. The optimum added contents used in this research were concluded from the investigations on the rheology property, ageing resistance, crack resistance, *etc.* Table 1 presents the basic characteristics of the active carbon filler, and Table 2 concludes on the fundamental properties of the studied materials.

**Table 1:** Basic characteristics of active carbon.

Items	Values
PH	5.0–7.0
Surface area	1100 m^2^/g
Residual after dried	90%
Dissolved by hydrochloric acid	Less than 0.8%
Dissolved by ethanol	Less than 0.2%

**Table 2:** The general performance of the evaluated materials.

Property	PJ90	PJ90 (4% SBS + 4% C)
Softening point (°C)	45	70
Ductility (15 °C) (cm)	>120	>120
Penetration (25 °C) (0.1 mm)	75.6	56.7

### 2.2. Principle of TG-MS Test

Thermogravimetry analysis studies the mass changes of a sample while it is subjected to a controlled temperature program [14]. However, it is only a quantitative thermal technique. It gives no direct chemical information. Mass spectrometry is an analytical technique that produces the spectrum of the masses of the molecules [15,16]. They might be combined together to characterize the additional chemical information. The spectrum can be used to illustrate the masses of molecules and to distinguish the structure and chemical properties of different molecules.

**Figure 1:**
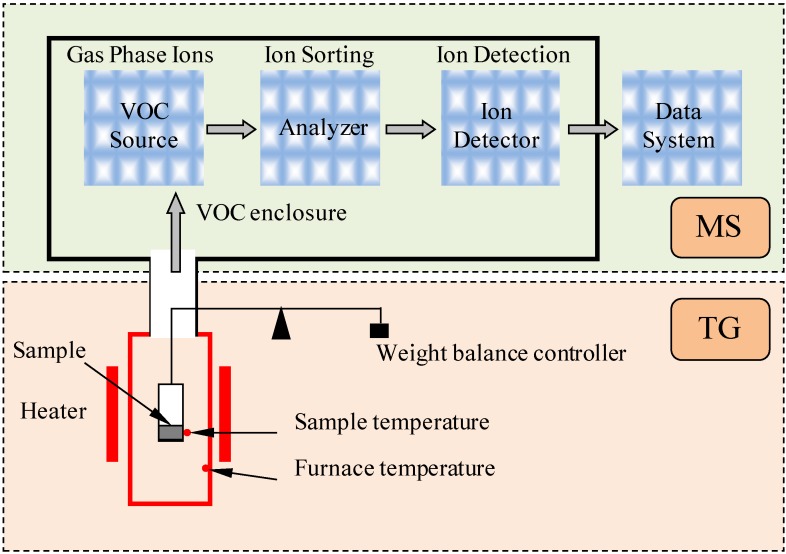
Principal of thermal gravimetric analysis-mass spectrometry (TG-MS) test and analysis program.

**Figure 2:**
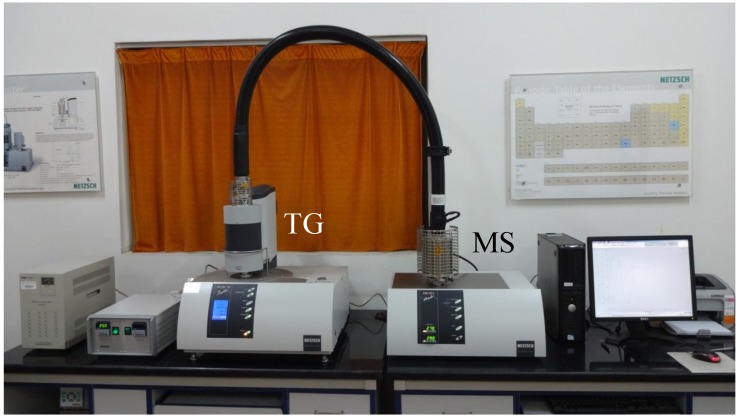
The TG-MS instrument used for characterizing volatile organic compound (VOC) emissions.

Thermal gravimetric analysis-mass spectrometry (TG-MS) is an analytical method that combines the features of thermogravimetry and mass spectrometer to identify different volatile products during a weight lost process with one test sample. Applications of TG-MS include weight detection, temperature investigation and identification of volatile emissions. TG-MS is therefore employed to analyze the bitumen VOC emissions, both qualitatively and quantitatively, in this research. Figure 1 introduces the principal of the TG-MS test and analysis program, and Figure 2 presents the TG-MS instrument.

When the VOC emissions go through a mass spectrometer, it then separates the ions according to their specific mass-to-charge ratio and records the relative amount of each ion type. Thus, a mass spectrum of the molecule can be produced. As Figure 1 shows, the collected VOC source ions will be resolving into their characteristics mass components according to their mass-to-charge ratio at the analyzer step. Then, the ion detector will detect the ions and record the relative amount of each of the resolved ionic species.

The STA 449 F3 Jupiter (TG) and QMS403C quadrupole mass spectrometer (MS), produced by NETZSCH (Altenstadt, Germany), were employed to analyze the chemical components during VOC emission from bituminous materials. The specimen was heated up to 300 °C in the TG instrument with the temperature increasing at a rate of 10 °C/min. In the meantime, MS was conducted to characterize the molecular weight and volatile speed of VOC emissions.

### 2.3. Ultraviolet-Visible Spectroscopy Test

Ultraviolet-visible spectrophotometry is a method to formulate the absorption spectroscopy in the ultraviolet-visible spectral region. It is widely employed to conduct the identification and measurement of organic and inorganic compounds in a wide range of products and processes. The Beer–Lambert law states that the absorbance of a solution is directly proportional to the concentration of the absorbing species in the solution and the path length. Thus, for a fixed path length, UV-Vis spectroscopy can be used to determine the concentration of the absorber in a solution, when the calibration curve has been determined to express the relation between absorbance and concentration.

The calibration curve can be determined by detecting the absorbance of incident monochromatic light at different wavelength when passing through solutions with a fixed concentration of the absorbing specimens. Based on our previous research [17], ultraviolet light with a wavelength of 288 nm was used to characterize the VOC emissions. The calibration curve was first defined and then used to determine the emission amount from bituminous materials.

**Figure 3:**
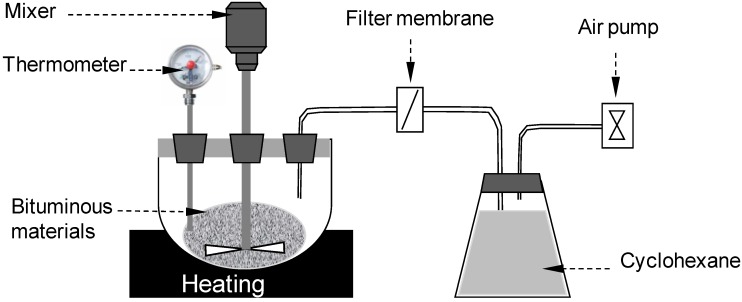
VOC generating and collecting device for the ultraviolet-visible spectroscopy test (UV-Vis) analysis.

A diagrammatic sketch of the VOC generating and collecting device for UV-Vis analysis is shown in Figure 3. The air pump (Airchek 2000 sampler, SKC Inc., Eighty Four, PA, USA) was connected at the end of the experimental system to create negative air pressure, leading to air flow from the specimen container to the solution container. The glass fiber filter membrane is first to filter the heavy components from the bituminous materials before the air goes through the cyclohexane, which was used as the solution to collect VOC emissions.

The bituminous material was heated to 180 °C to accelerate the VOC emission speed. The sampling flow was 500 mL/min, and the sampling time was 15 min. A UV-1601 visible spectrometer, produced by Japanese Shimadzu, was used in this research to investigate the amount of VOC components.

### 2.4. Rheological Property Analysis

**Figure 4:**
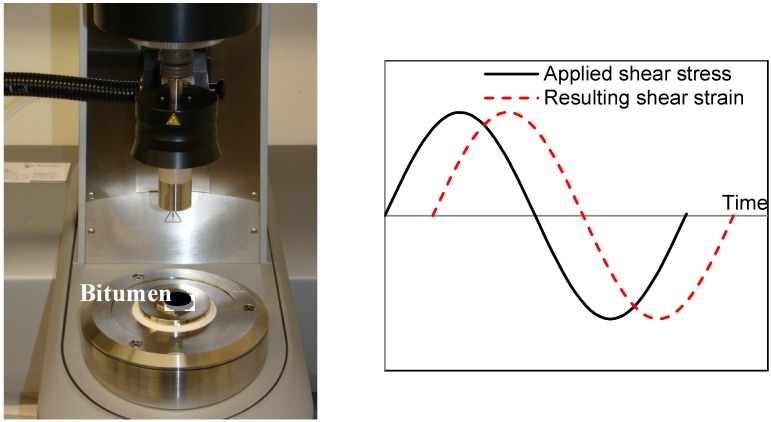
The dynamic shear rheometer (DSR) instrument and the applied sinusoidal signal.

A dynamic shear rheometer (DSR), produced by Anton Paar Austria (Graz, Austria), was used to evaluate the rheological properties at varied temperatures and frequencies. Temperature sweep tests within a wide range of frequencies and frequency sweep tests at different temperatures were conducted separately. The sinusoidal signal as Figure 4 shows was applied on the bituminous specimen. A spindle with an 8-mm diameter was used for lower temperature ranges from 5 °C to 30 °C, while a 25-mm spindle was used for higher temperature ranges from 30 °C to 80 °C. The phase angle and complex shear modulus response of tested specimen were then analyzed.

## 3. Results and Discussions

### 3.1. Residual Volatile Speed

Residual volatile speed was first introduced to characterize the changing of VOC emission before and after adding inhibitors into bitumen. The residual volatile speed was defined as the ratio between the VOC volatile speed of bituminous material modified with inhibitors and the VOC volatile speed of non-modified bituminous material:

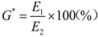
(1)
where G^*^ is the residual volatile speed, *E*_1_ is the VOC volatile speed of bituminous material modified with inhibitors, while *E*_2_ is the VOC volatile speed of bituminous material without modified with inhibitors.

When G^*^ > 1, the added VOC inhibitor is unsteady and cannot inhibit the VOC emission at the evaluated temperature. Instead of decreasing the volatile speed, the speed would be increased by using such an inhibitor. When G^*^ < 1, the contribution of the added inhibitor was positive, and the VOC volatile speed can be decreased to a certain level.

Normal octane and dichloroethane, which can cause damage to the nervous system, liver, kidneys and lungs and may cause cancer, are reported as the two main volatile components from bituminous materials during their heating and service period. Naphthalene is concerned as the most volatile PAH (Polycyclic aromatic hydrocarbon) from bituminous materials [18]. It is also well known that the naphthalene compounds and phenanthrene compounds are the main components of VOC, which are hazardous to human health and the environment. Therefore, these four types of gas in the VOC emissions from bituminous materials, including dichloroethane (molecular weight 98, C_2_H_4_Cl_2_), normal octane (molecular weight 114, C_8_H_18_), naphthalene (molecular weight 128, C_10_H_8_) and methylphenanthrene (molecular weight 191, C_15_H_12_), were collected and analyzed. Table 3 summarizes the fundamental information for these four analyzed VOC gas.

**Table 3:** Information for the four analyzed VOCs.

Volatile Type	Molecular Type	Main Characteristics
Naphthalene	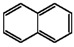	The most volatile components from bituminous materials, may cause cancer
Dichloroethane	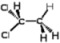	The two main volatile components from bituminous materials, toxicity to humans and aquatic organisms
Normal octane	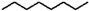	
Methylphenanthrene	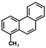	Main components of VOC, toxicity to humans and aquatic organisms

**Figure 5:**
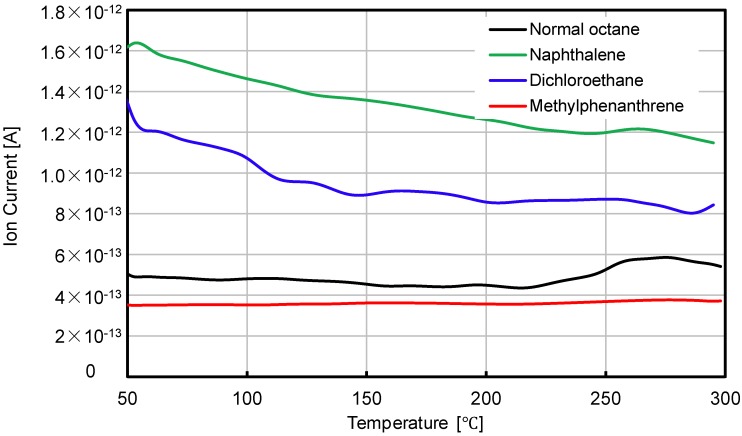
VOC volatile speed of non-modified bituminous material.

Figure 5 illustrates the VOC volatile speed of non-modified bituminous material. The emission of naphthalene and dichloroethane mainly happened at the beginning stage of the test, although at the beginning, the conditional temperature was lower.

**Figure 6:**
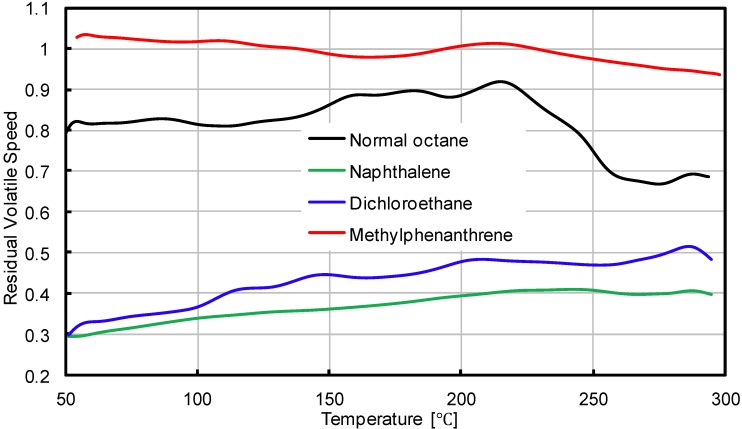
Residual volatile speeds of four types of gas.

Figure 6 compares the residual volatile speed of the four analyzed VOC gases. It is clear that the combined introduction of SBS and active carbon filler into bituminous materials can significantly decrease the VOC emission speed, while the decreasing influence is different on different types of gas. At the very beginning stage of the test, the volatile speed of naphthalene can be decreased by more than 70% when the SBS and active carbon filler were added. The emission speed of dichloroethane decreased by more than 60% and about 20% for normal octane. With the increasing test temperatures, the inhibitory effect changed. With naphthalene and dichloroethane, the inhibitory effect decreased with the increasing of temperature; while with normal octane and methylphenanthrene, the influence of temperature on the inhibitory effect is not as significant as that compared to the other two types of VOC gas.

### 3.2. Ultraviolet-Visible Spectroscopy Test (UV-Vis) Test Analysis

The evaluation of VOC amount was based on the linear relationship between the VOC quality of the absorption solution and its absorbance value. Therefore, the relationship between the absorbance of the standard bituminous specimen and the amount of VOC emission was first finalized.

#### 3.2.1. Calibration Curve

**Figure 7:**
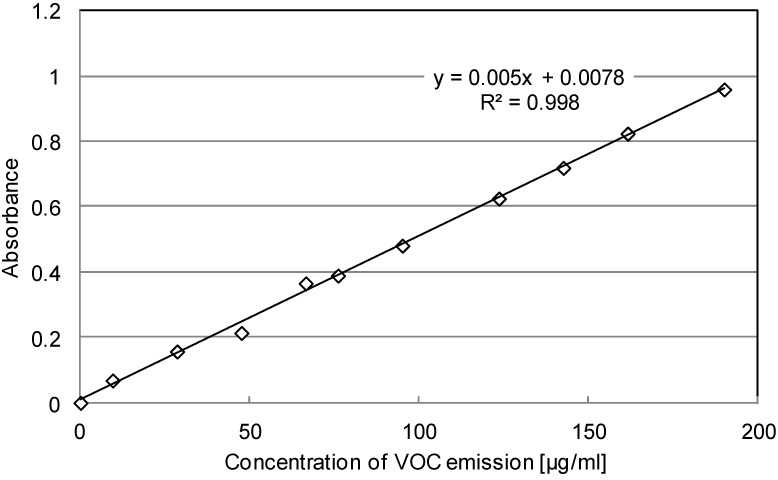
Mathematical solvers for the calibration curve.

A certain amount of cyclohexane solution was used to absorb different known amounts of VOC, resulting in solutions with a variety of VOC concentrations. These treated solutions were than tested with UV-Vis. At the end, the relationship between VOC concentration and absorbance can be concluded, as shown in Table 4. Figure 7 presents the calibration curve.

**Table 4:** Relationship between the absorbance and concentration of VOC emission.

No.	Absorbance (*Y*)	Concentration of VOC (μg/mL, *X*)
1	0	0
2	0.0686	9.4949
3	0.1573	28.4848
4	0.3897	47.4747
5	0.4813	66.4646
6	0.6254	75.9596
7	0.7192	94.9495
8	0.8242	123.4343
9	0.9594	142.4242
Mathematical Solver		*Y* = 0.005*X* + 0.0078 (*Y* = *aX* + *b*, *R*^2^ = 0.998)

Figure 7 illustrates that the relation between absorbance and the amount of VOC emission shows a good linear correlation. Such a linear correlation can be expressed with the function presented in Table 4. Two constant parameters, “*a* = 0.005” and “*b* = 0.0078”, were used to define the calibration curve.

#### 3.2.2. UV-Vis Data

Based on the calibration curve, the UV-Vis test was conducted with PJ90 and modified bituminous material. The quantity of VOC emissions from every gram of bituminous material can be calculated according to this equation (μg/g):

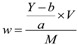
(2)
where *M* is the weight of tested bituminous material (g), *a* and *b* are the constant parameters of the calibration curve, *Y* is the absorbance value of the test sample and *V* is the volume of cyclohexane that was used to dissolve VOC (mL).

In this research, this volume was fixed at 18 mL, while only 3.5 mL of cyclohexane with dissolved VOC were used for UV-Vis evaluation. The test results were presented in Table 5. The VOC was collected within 15 min when the bituminous material was heated at 180 °C.

It is obvious from Table 5 that under the same temperature condition, the amount of VOC emission from PJ90 bituminous material was much higher than that from bituminous material modified with inhibitors. The analysis shows that 7.886 μg/g of VOC emissions volatilized from PJ90, while only 3.63 μg/g of VOC emissions were found from the modified bituminous material. The combination of using SBS and active carbon filler as inhibitors can prevent more than 50% of VOC molecules from volatilization.

It is believed that the SBS modifier with a physical cross-linking structure can prevent the small molecules in the bituminous materials from escaping to the surface. In the meantime, an ultra-fine granular additive, such as activated carbon, has a very remarkable higher surface energy than traditional fillers. Such characteristics could absorb small molecules, resulting in lower VOC emission.

**Table 5:** Quantity values of VOC emission from bituminous materials.

Parameter	PJ-90	PJ90 (4% SBS + 4% C)
*M* (g)	935.48	1190.94
*Y*	2.0569	1.2087
*V* (mL)	18	18
Concentration of VOC in cyclohexane (μg/mL)	409.82	240.18
Total amount of VOC (μg)	7376.76	4323.24
*W* (μg/g)	7.886	3.63
VOC decrement percentage (%)	53.96	

### 3.3. Rheological Behaviors

The TG-MS test results strongly proved that adding functional modifiers into bituminous materials can prevent the VOC from emission. Besides this remarkable achievement of inhibiting VOC, if these modifiers can maintain the material behavior of bituminous materials for pavement purposes in the meantime, it would be very helpful to design environmentally-friendly bituminous materials.

#### 3.3.1. Temperature Sweep

**Figure 8:**
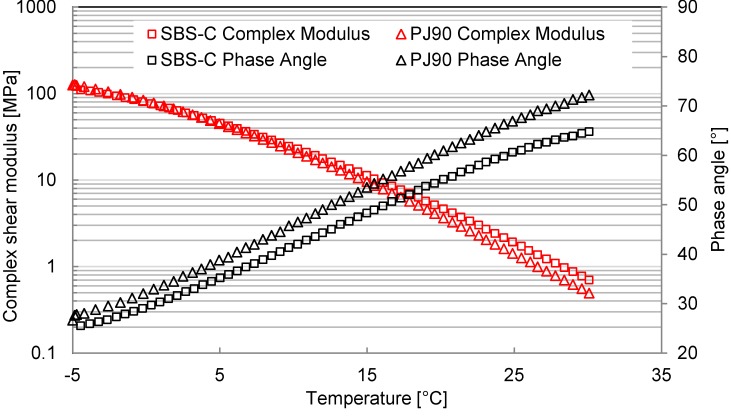
Phase angle and modulus curves at the lower temperature range.

**Figure 9:**
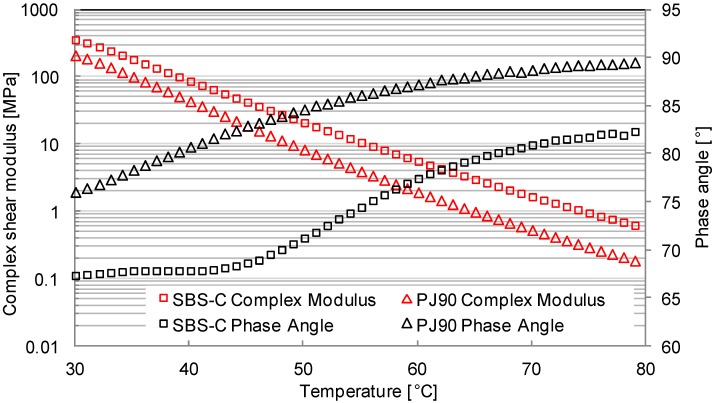
Phase angle and modulus curves at the higher temperature range.

Figure 8 and Figure 9 compare the differences of phase angle and the shear complex modulus between bituminous materials with and without additives of the VOC inhibitor, at a loading frequency of 10 rad/s.

With the increasing of the test temperature, the phase angle of both bituminous materials increased, while the complex shear modulus decreased. This is because the bituminous material is a viscoelastic material. It has a viscous domain behavior at higher temperature, while at a lower temperature, it has an elastic domain behavior. The amount of viscous components in the bituminous material was increasing, resulted from the increase of temperature. The state of bituminous materials will transform from an elastic state to a viscous state when the temperature is increased. This process will then accelerate the movement of small molecules.

The additives of SBS and active carbon filler will decrease the values of the phase angle, while increasing the values of the complex shear modulus. This indicates that the added inhibitors can enhance the material behavior contributed by the elastic component, as well as improve the high-temperature resistance. Furthermore, both the slopes of the modulus curve and the phase angle curve for modified bituminous materials are smaller than those values for the PJ90 bituminous material. Such a slope represents the temperature susceptibility of the tested materials. Therefore, it can be concluded from Figure 8 and Figure 9 that the modified bituminous material is not as sensitive to temperature as the PJ90 material is. The combination of adding 4% SBS and 4% active carbon filler into bituminous materials could decrease the temperature susceptibility.

Under a lower temperature condition, the differences of the phase angle or the complex shear modulus of two kinds of bituminous materials were smaller than the differences at higher temperature conditions. This proved that the influence of SBS and active carbon filler on the bituminous behavior is more significant at higher temperature ranges than at lower temperature ranges. The VOC emission speed increases with the increase of the conditioning temperature. Such a phenomenon will make the significant influence of SBS and active carbon filler at a higher temperature range greater.

#### 3.3.2. Frequency Sweep

Frequency sweep tests were performed by means of an applied sinusoidal strain signal on bituminous materials at fixed testing temperatures under a wide frequency range from 0.1 rad/s to 400 rad/s. The fixed test temperatures were −10, 0, 10, 20, 30, 40, 50 and 60 °C. Master curves of the complex shear modulus and the phase angle of the two evaluated bituminous materials were constructed at the reference temperature of 20 °C, with the data from the frequency sweep test.

**Figure 10:**
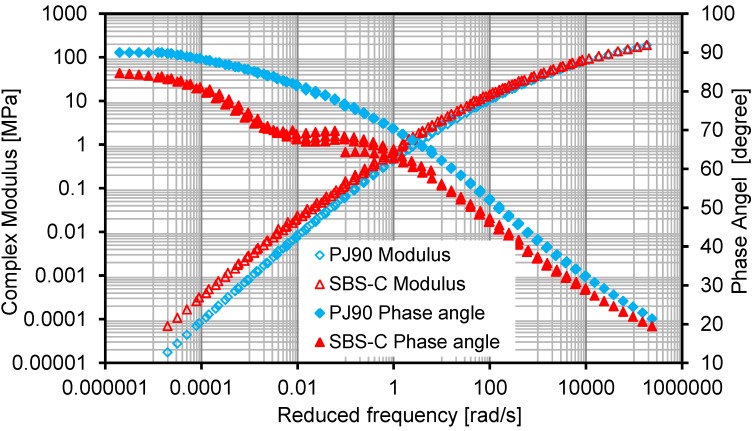
Master curves of two kinds of bituminous materials.

The master curves can be used to analysis the material behavior at a wide range of loading frequencies. Based on the time-temperature superposition principle [19], the material behavior at a lower frequency range can be theoretically transferred to represent the material behavior at a higher temperature condition, and *vice versa*. Figure 10 proved that the introduction of SBS and active carbon filler can slightly increase the modulus values, but significantly decrease the values of the phase angle. Furthermore, such influences resulting from SBS and active carbon filler are more obvious at a lower frequency range than at a higher frequency range. At a frequency higher than 200 rad/s, the influence on the modulus is negligible, while the differences on the phase angle are also very small.

Research results from both the temperature sweep test and frequency sweep test indicated that the combination of adding 4% SBS and 4% active carbon filler into bituminous materials has a significant contribution to improving the deformation resistance behavior at higher temperatures.

## 4. Conclusions

The VOC emission and material behaviors were analyzed and compared in this research to investigate the possibility of adding SBS and active carbon filler into bituminous materials to develop environmentally-friendly materials. Thermal gravimetric analysis-mass spectrometry and ultraviolet-visible spectroscopy testing were employed to characterize the VOC emission process. The temperature sweep test and frequency sweep test were conducted to evaluate the rheological properties of bituminous materials with and without SBS and active carbon filler as VOC inhibitors. Based on the research results discussed, the following conclusions can be finalized:

1. TG-MS, a combination of thermogravimetry and mass spectrometry can be used to identify different VOC components from bituminous materials during a weight lost process.
2. The combined introduction of SBS and active carbon filler into bituminous materials can significantly decrease the VOC emission speed, while the decreasing influence is different on different types of gas. Such an inhibitory effect resulting from SBS and active carbon filler is temperature related.
3. Under the same temperature condition, the amount of VOC emission from PJ90 bituminous material was much higher than that from bituminous material modified with inhibitors. The combination of using SBS and active carbon filler as inhibitors can prevent more than 50% of VOC molecules from volatilization.
4. The combined introduction of SBS and active carbon filler into bituminous materials can not only lower the VOC emission speed and amount, but also improve the deformation resistance behavior at a higher temperature, which was proven by means of a temperature sweep test and a frequency sweep test.
